# Know your neighbor: The impact of social context on fairness behavior

**DOI:** 10.1371/journal.pone.0194037

**Published:** 2018-04-11

**Authors:** Neelanjan Sircar, Ty Turley, Peter van der Windt, Maarten Voors

**Affiliations:** 1 Center for Policy Research, New Delhi, India; 2 Marriott School, Brigham Young University, Provo, Utah, United States of America; 3 Social Science Division, New York University Abu Dhabi, Abu Dhabi, United Arab Emirates; 4 Development Economics Group, Wageningen University, Wageningen, the Netherlands; Middlesex University, UNITED KINGDOM

## Abstract

Laboratory experiments offer an opportunity to isolate human behaviors with a level of precision that is often difficult to obtain using other (survey-based) methods. Yet, experimental tasks are often stripped of any social context, implying that inferences may not directly map to real world contexts. We randomly allocate 632 individuals (grouped randomly into 316 dyads) from small villages in Sierra Leone to four versions of the ultimatum game. In addition to the classic ultimatum game, where both the sender and receiver are anonymous, we reveal the identity of the sender, the receiver or both. This design allows us to explore how fairness behavior is affected by social context in a natural setting where players are drawn from populations that are well-acquainted. We find that average offers increase when the receiver’s identity is revealed, suggesting that anonymous ultimatum games underestimate expected fair offers. This study suggest that researchers wishing to relate laboratory behavior to contexts in which the participants are well-acquainted should consider revealing the identities of the players during game play.

## Introduction

Behavioral games have become popular tools to make inferences about hard-to-measure preferences. Workhorse games include dictator games to understand altruistic preferences, ultimatum games to measure preferences for fairness, and public goods games to learn about cooperative behavior. These sorts of inferences cannot be easily discerned from standard survey or secondary data sources. However, the use of experiments remains controversial because participants are typically studied in isolation of their social context. As such, critics argue that behavioral games are artificial and the resulting inferences necessarily flawed. In this paper, we explore how laboratory behavior may be impacted by incorporating contextual information among populations where individuals are well-acquainted with each other.

Behavior in laboratory games departs from standard human interaction in a number of key ways. Typically, participants do not have direct interaction with their counterparts, and are often unacquainted with each other. In addition, during game play the identities of the participants are not revealed. The original protocol for the dictator game, for example, entailed informing the dictator that the receiver was behind a closed door to ensure anonymity [1]. In such a setting, no attributes of the receiver, such as personal characteristics or information obtained from previous interactions, can affect the decision of the dictator. Most interactions in daily life, however, occur between individuals that are well-acquainted. Factors such as knowledge of a counterpart’s personal characteristics, previous and possible future interactions, and the social networks in which the individuals are embedded—i.e. the “social context”—are often key drivers of individual behavior [2].

In this paper, we investigate the role of social context in the ultimatum game. In particular, we randomly allocate 632 individuals (grouped randomly into 316 dyads) from small villages in Sierra Leone to four versions of the ultimatum game. The first version is the classic version of the ultimatum game, where both the sender and the receiver are anonymous. That is, their identities are not revealed to each other. In addition, we randomize sender-receiver dyads to conditions where 1) the identity of the receiver is revealed to the sender but the sender is unknown to the receiver; 2) the identity of the sender is revealed to the receiver but the receiver is unknown to the sender; and 3) both the identities of the sender and the receiver are revealed to each other. This design allows us to explore the causal impact of varying the extent to which social context matters for experimental contributions.

In rural Sierra Leone, like in many rural communities around the world, economic and social interactions occur within local, dense social networks, among populations with individuals that know each other well [3]. This implies that real-life interactions between study participants are unlikely to be driven by abstract notions of fairness, but rather by deeply contextualized understandings of fairness that are embedded in daily village life and formed by day-to-day bargaining between villagers over food, land, and risk. Furthermore, studying norms of fairness is particularly important in the Sierra Leone context, where grievances from unfair behavior by rural elites have been suggested as a major contributing factor to the 1991-2002 civil war [4, 5].

We find that in versions of the ultimatum game in which the receiver’s identity is revealed, the average offer increases. This suggests that anonymous ultimatum games underestimate expected fair offers and is consistent with other social behaviors, such as altruism [6] and trust [7].

This study addresses a key question concerning the use of classic experimental protocols to study human behavior. Many behavioral games abstract from the social context of human behavior to estimate underlying preferences. As a result, inferences drawn from these games are unlikely to translate directly to situations where participants know each other. In real life, an individual’s behavior is very different depending on the counterpart with whom interaction takes place, whether these counterparts are fellow villagers in Sierra Leone or friends and colleagues in New York City. Our study therefore explores an important link between nuanced information from the laboratory and the real world.

The rest of the paper is organized as follows. The subsequent section reviews the literature on the role of social context in ultimatum games. We then introduce our sample of participants in Sierra Leone, the experimental game variations and empirical strategy. We next present the results and close with a discussion.

## Ultimatum games and social context

Measuring social preferences using laboratory-style experiments broadly encompasses investigations into trust, reciprocity, fairness, altruism and the propensity for cooperation [8]. The workhorse experiment to understand fairness behavior is the ultimatum game, first studied in [9] (see [10] for a detailed explication). In the ultimatum game, the “sender” splits a fixed sum of money between herself and the “receiver”. The receiver subsequently decides to accept or reject the offer. If the offer is accepted, both players receive the proposed allocation. If rejected, both players receive nothing. In the classic version of this game, the sender and the receiver are not given any information about each other and are often unacquainted with each other outside the game. If both the sender and receiver were only materially self-interested, one would expect offers in the ultimatum game to be nearly zero and such offers to be accepted by the receiver. Empirically, however, one finds that senders usually offer 25-50% of the endowment, and offers less than 20% are typically rejected by the receiver [8]. This behavior is often taken as evidence that players are not purely materially self-interested and that the receiver may “punish” the sender for a proposed offer that is deemed to be unfair.

### The role of social context

There are compelling reasons why behavioral games use anonymity and/or play with subjects that are unacquainted with each other. It allows researchers to isolate underlying behavioral preferences without contamination by social considerations. However, for many such studies, the real-world behaviors they are designed to measure, such as altruism, trust and fairness, are inextricably linked to, or embedded in, a social context. Abstracting from the social context implies ignoring a large part of what drives behavior. Indeed, Levitt et al. [2] highlight that real-life behavior is influenced by not only monetary calculations but also by several other factors including: (i) the stakes of the game; (ii) the presence of moral and ethical considerations; (iii) the extent to which one’s actions are scrutinized by others and the nature of such scrutiny; (iv) the subject pool of respondents; and (v) the context in which the decision is embedded.

One common approach to introduce some social context is the “attribute-based” behavioral game. In such a laboratory game, certain attributes of another player, like ethnicity or gender, are revealed but the rest of the interaction is either anonymized or the participants are otherwise unacquainted with each other. The intuitive empirical interpretation of such studies is that it measures the extent of the behavior between random strangers who can only discern the attribute under question. It is natural to use this type of game to characterize “statistical discrimination,” the extent to which all beliefs that can be gleaned from an attribute predicts differences in outcomes. These ideas have been applied to, for instance, the housing or labor market, where employers and landlords must sort through large numbers of applicants with minimal information. Such studies, however, provide little empirical leverage on contexts where individuals regularly interact and have a large amount of contextual information. Furthermore, observed differences in laboratory behavior may not be due to some experimentally manipulated revelation of a single attribute but rather a function of more complex interactions that were not manipulated by the experimenter [11].

This study takes these arguments to their logical ends. Because the social behaviors we seek to measure—fairness among individuals that know each other—are likely a function of complex interactions between individuals, we do not manipulate a single attribute. Rather, we manipulate whether the sender and/or receiver are revealed to each other in the game. This paper thus estimates the impact of including various levels of contextual information in an ultimatum game. The ultimatum game is typically used to deduce abstract principles of fairness, where these principles emanate from what the receiver deems to be a “fair” allocation. Our contribution is to understand how these principles of fairness change as a function of information about the social context.

### Related studies

In S1 Supporting Information we present an overview of the fifteen most cited ultimatum game studies. These studies show that behaviors related to fairness depend on many factors, such as player characteristics (e.g. gender) and the context of interactions, like the stakes involved in the game [12]. In thirteen of the fifteen studies, both the sender and receiver play anonymously, and the games are typically conducted between university students. Notable exceptions are Solnick et al. [13] and Charness et al. [14]. The first study reveals the identity of the receiver and the sender through a photograph to learn about the influence of physical attractiveness on the bargaining process. However, the researchers explicitly recruit two non-overlapping samples from different universities to ensure that players are unacquainted with each other. In Charness et al. [14], participants are given the family name of their counterparts, but the authors are careful to note, “Participants from two different universities are used to ensure that this is the only additional information they receive (that is, they do not know their counterparts personally)” (page 30). By keeping the sender and/or receiver anonymous, or by revealing identities of unacquainted individuals, factors such as knowledge of a counterpart’s personal characteristics, previous interactions, beliefs about future relations, and the social networks in which they are embedded are precluded from driving a player’s behavior.

Table 1 presents ten recent papers that use ultimatum, dictator or trust games to investigate the effects of varying the anonymity of players. Both Burnham [15] and Bohnet et al. [16] conduct a set of dictator games where the identities of the senders and receivers are revealed to each other. In the first study, the identities were revealed through a photograph, and in the second study subjects were asked to stand up and look at each other in silence before they made their decision anonymously. In both studies, the subjects were students unacquainted with each other outside of the game. Bohnet et al., for example, explicitly notes, “The subjects were recruited during their second week at the university. Therefore most students did not know each other before the experiment (page 47 in [16]).”

**Table 1:** Experimental studies that vary anonymity*.

Study	Type	Location	Sender	Receiver	Sample	Participants
This study	UG	LF	R	R	K	Rural villagers in Sierra Leone
Burnham [15]	DG	L	R	R	U	Subjects at University of Arizona
Bohnet et al. [16]	DG	L	R	R	U	Students at University of Zurich
Binzel et al. [7]	TG	LF	A	R	K	Members housing areas in Cairo
Dufwenberg et al. [17]	DG	L	R	A	K	Students at Stockholm University
Brañas-Garza et al. [18]	DG	L	A	R	K	Students at University of Granada
Brañas-Garza et al. [19]	DG	L	A	R	K	Students at University of Granada
Cobo-Reyes et al. [20]	CG	L	A	R	K	Students at University of Granada
Kovářík et al. [21]	DG	L	A	R	K	Students at University of Granada
Leider et al. [22]	DG	LF	R	R	K	Students at Harvard University
Ligon et al. [23]	DG	LF	R	R	K	Rural villagers in Paraguay

* Under ‘Type’, “UG”, “TG”, “DG” and “CG” indicate ultimatum, trust, dictator and coordination games, respectively. In column ‘Location’, “L” indicates laboratory games, whereas “LF” are lab-in-the-field experiments. In columns ‘Sender’ and ‘Receiver’, “A” (“R”) indicates whether the player is anonymous or revealed. Finally, the column ‘Sample’ indicates if the subjects are known to each other (“K”), or not (“U”).

In contrast, Binzel et al. [7] and Dufwenberg et al. [17] conduct, respectively, a set of trust and dictator games in which the participants are well-acquainted with each other. However, both of these studies reveal only one of the players; in the first study, only the receiver is revealed, and in the second study, only the sender is revealed. Brañas-Garza et al. [18] [19], Cobo-Reyes et al. [20] and Kovářík et al. [21] also conduct games with well-acquainted participants. As part of the design, participants were asked to list their close friends. Senders knew that the receiver would be a friend drawn randomly from their own list. In all four studies, the aim was anonymity of the receiver’s identity, except for their friendship status. Brañas-Garza et al., for example, explicitly note, “Although [senders] know whether the recipient is a friend or not, they ignore the identity, so between-subjects anonymity is preserved.” (page 173 in [19]). Only in the rare instances where senders listed solely one friend, did the sender know the identity of the receiver.

Closest to our study are Leider et al. [22] and Ligon et al. [23]. The first conducted online dictator games among Harvard University undergraduates from the same dormitory and decomposes the motivation for sharing into: (i) altruism towards a randomly selected stranger; (ii) altruism towards a friend; and (iii) altruism motivated by the prospect of future interaction (by revealing the sender). Ligon et al. [23] conducted a set of dictator games with households in fifteen villages in rural Paraguay. Dictators were asked to play four games that varied whether the dictator was anonymous or revealed, and whether the recipient was randomly selected and remained anonymous or was purposefully selected by the dictator and thus revealed to the dictator. In this paper, we depart from these two studies in two important ways. First, we relax anonymity in a setting where players are well-acquainted with each other, and the sender cannot choose the receiver. In our setup, senders and receivers are randomly matched, and each of the dyads are randomly assigned to one of four treatment groups. We therefore measure behavior between pairs of individuals that can be compared to the classical game, since the composition of dyads is not affected by selection due to random assignment. Furthermore, we can isolate the causal effect of introducing a common social context on laboratory behavior by revealing identity between individuals well-acquainted with each other. Second, this paper assesses the impact of relaxing anonymity on behavior in the ultimatum game. While Leider et al. [22] and Ligon et al. [23] have investigated the impact of relaxing anonymity on altruism and trust, there is less understanding of its impact on norms of fairness.

## Material and methods

We conducted a set of laboratory experiments in 35 small and remote communities in rural Sierra Leone in 2010. These communities lie close to the Gola Rainforest National Park in Eastern Sierra Leone. Our research villages are small with an average population size of about 220 individuals divided over an average of 28 houses, implying that economic and social interactions between villagers is frequent. These numbers are in line with successive national censuses (1963, 1984, 2004). The reach of the government is limited, and formal, legal and political institutions are less important than the local institutions and social structures. Local elites, often appointed for life, have great authority to organize economic and social activity, including the power to raise taxes, mobilize labor, settle disputes and allocate resources such as land, labor and reproductive opportunities [5, 24, 25]. Community-level activities such as the organization of public goods projects, dispute settlement and collaborative efforts are governed by local institutions and social norms of cooperation [3, 26, 27]. Behavior and social exchange and interactions are governed by a deeply entrenched patronage relationship of interrelated gifts and obligations [28]. For example, Bulte et al. [29] show the importance of social context for trading behavior in Sierra Leone. In sum, we expect fairness behavior by individuals in rural Sierra Leone to be driven not only by individual preferences, but to a large extent by the social context. This characterization of the dense social networks of rural communities is not unique to Sierra Leone and is reflected in writings on gift exchange [30–32].

Furthermore, a careful understanding of norms of fairness is important in Sierra Leone. The society is governed by patron-client relationships, where villagers depend on a highly exclusionary set of traditional institutions if they want access to property or political rights. The system has historically created a large class of excluded, low status individuals (mostly migrants and young men). Some scholars of Sierra Leone argue that forced community labor, harsh fines imposed by chiefs, and lack of employment opportunities (all products of this system) created feelings of disenfranchisement and resentment among rural youth, which in turn was a major contributing factor to the 1991-2002 civil war [4, 33, 34]. For example, Humphreys et al. [35] show that the Revolutionary United Front (RUF) found significant popular support from students and farmers upset with the recurring marginalization and humiliation. There is evidence that grievances as a result of this unfair treatment by rural elite have persisted in the post-war period [5]. Note, however, that Voors et al. [36] find little evidence that grievances explain the onset or duration of violence during the civil war in Sierra Leone.

A total of 632 heads of household participated in our ultimatum games. Specifically, we grouped the participants into 316 randomly created dyads. We then randomly assigned each individual in the dyad to the role of sender or receiver. The senders were endowed with 5,000 Le (around 1.1 USD, about a day’s wage) and asked to offer a portion of their endowment to the receiver. The receiver could then accept or reject the offer. If accepted, the sender and receiver kept the division proposed by the sender; otherwise both received nothing. To test the effect of introducing social considerations to the ultimatum game we randomly allocated the dyads in the village to one of four versions: (i) both the sender and the receiver played anonymously (which we denote as AA); (ii) the identity of the receiver was revealed to the sender (AR), (iii) the sender was identified to the receiver, and the sender made her decision knowing that the receiver would know her name (RA), and (iv) the names of both the receiver and the sender were revealed to each other (RR). The script that was given to players after discussing the rules of the ultimatum game can be found in S1 Supporting Information. We note that when the sender, receiver or both are revealed, the sender and receiver may potentially interact and future economic interactions may be shaped by game play, capturing a realistic aspect of dyadic interactions not included in anonymized games.

### Ethics statement

We obtained verbal informed consent from the respondents because in rural Sierra Leone very few people can read or write. Research assistants went through an explanation of the research and asked respondents whether they understood and agreed to go ahead. Respondents were informed that they were not obliged to answer questions if they did not want to and were free to stop the interview at all times. The research assistant recorded the answer of the respondent and any remarks made on the survey form. We obtained Institutional Review Board (IRB) approval for this study and related protocols from the IRB of Njala University in Sierra Leone (#FWA00018924), and from the IRB of the University of Chicago (#H10076) where Ty Turley was affiliated during the whole period of data collection.

### Empirical strategy

Our empirical strategy is straightforward. We compare the average levels of the offers across the four conditions. To make causal claims, our analysis relies on complete randomization of participants to experimental conditions within each village. This guarantees that the average characteristics of the study participants for each of the four conditions are similar in expectation [37]. In S1 Supporting Information we compare a number of key characteristics across the four conditions. Our players are on average 44 years old, predominantly male, Muslim, and ethnically Mende. Mean family size is six, and half of the sample is from chiefly families (families that can stand for the chieftaincy). We find that while some differences are significant, there are no strong systematic differences across these four conditions, which we would expect as a result of our randomization strategy.

## Results

We start by looking at descriptive statistics about offers in the ultimatum game. Close to all senders made a positive offer, with only three subjects offering zero. In total, only eight offers were rejected (the average offer in that case was 1,438 Le), suggesting that senders were largely successful in internalizing how little the receiver would be willing to accept before rejecting the offer. The distribution across treatment arms is as follows: AA:1, AR:0, RA:2, RR:5. In a set of ultimatum games conducted among non-integrated Spanish gypsies, Brañas-Garza et al. [38] find that receivers often accepted zero offers for reasons of solidarity. As we have discussed above, fairness is an important driver of behavior in Sierra Leone. Unfortunately, because few offers were rejected in our study we cannot explore the behavior of receivers further. The average offer is 37% of the endowment, or 1,840 Le.

**Fig 1:**
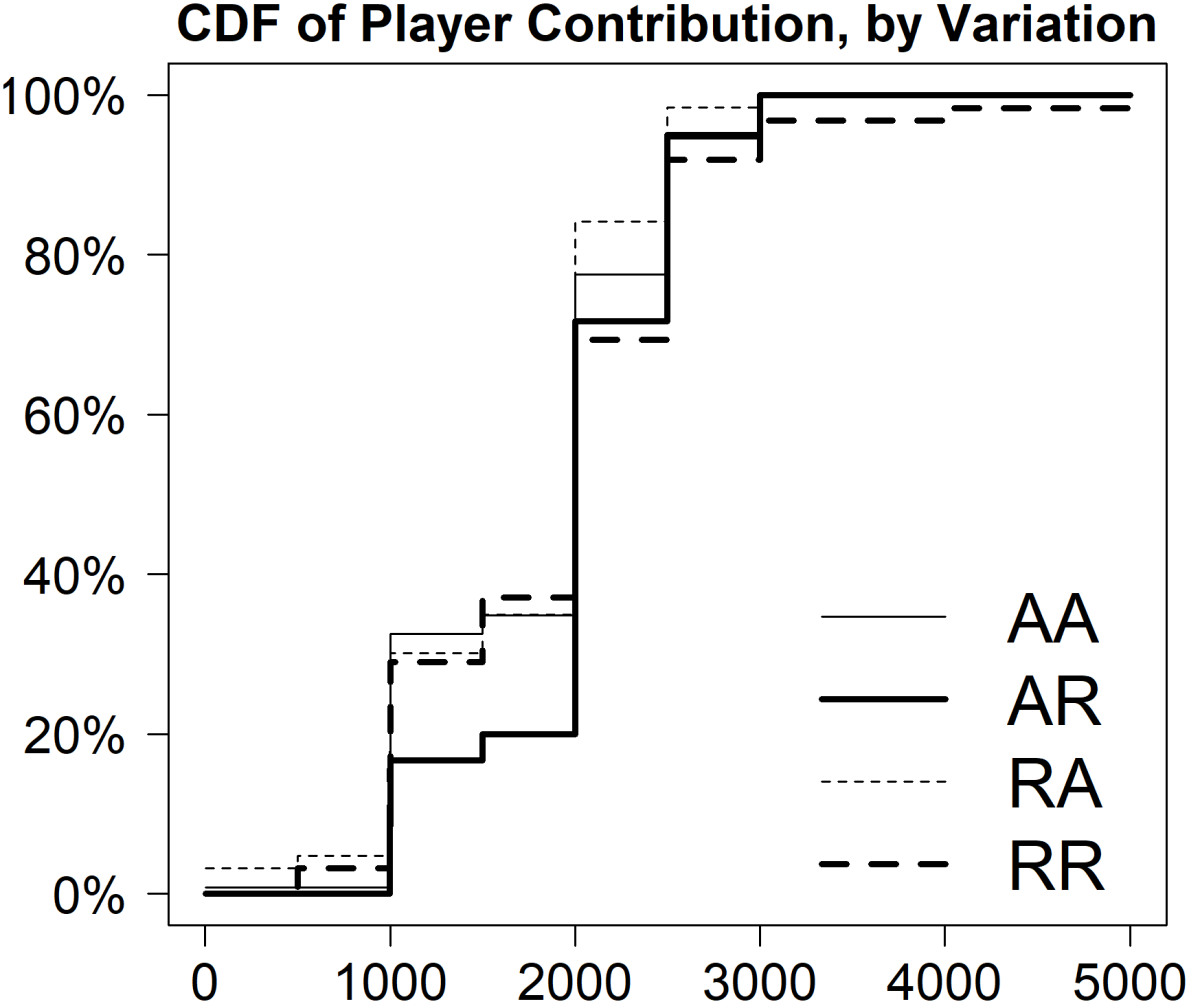
CDF of offers by experimental condition.

Focusing on our treatment variations, Fig 1 presents the cumulative distribution function (CDF) of contributions across the four conditions. Data is based on a total of 314 senders: 129 (AA), 60 (AR), 63 (RA) and 62 (RR). Two observations missed information on offer size for a total of 314 dyads in our reported sample. There is more variance in offers in the RR condition, as compared to the others. The RR condition has the highest proportion contributions of 2,000 Le or less and the longest tail (i.e. while the other treatment arms have maximum values of 3,000 Le, the largest contribution in RR is 5,000 Le).

Table 2 presents the main results. We report standard errors in parentheses. The fully anonymous treatment (AA) is set as the baseline category. P-values for across treatment arm comparisons (AR vs. RA; AR vs. RR; and RA vs. RR) are included. We present results for three increasingly more demanding model specifications. In Column (1) we cluster the error at the village level, taking into account that our data is clustered by village. In Column (2) we control for village level fixed effects, our level of randomization. In Column (3) we control for village level fixed effects and individual characteristics (the same as used for our balance tests (see S1 Supporting Information). When including control variables, the number of observations decreases to 298 due to missing data. Specifically, for Column (3) we estimate:

$$\begin{array}{cc} & Y_{i}=\beta_{1}{\text{AR}}_{i}+\beta_{2}{\text{RA}}_{i}+\beta_{3}{\text{RR}}_{i}+\gamma I_{i}+\alpha_{v}+\epsilon_{i}, \end{array}$$ (1)

where *Y*_*i*_ is the offer by sender *i*. *AR*, *RA* and *RR* are dummy variables and equal to one if sender *i*’s dyad received the respective treatment, and zero otherwise. *I*_*i*_ is a vector of individual characteristics, *α*_*v*_ are village level fixed effects and *ϵ*_*i*_ is a normally distributed error term. Data is publicly available and the code used to run the regressions can be found online.

**Table 2:** Mean offers by experimental condition*.

	Mean Offer		
	(1)	(2)	(2)
AR	188.760**	269.505**	270.170**
	(92.160)	(116.856)	(119.185)
RA	-72.351	-0.130	4.489
	(101.690)	(114.976)	(117.816)
RR	103.813	172.799	167.957
	(140.931)	(115.367)	(120.615)
Mean offer in AA	1794.57	1794.57	1794.57
Observations	314	314	298
R^2^	0.0185	0.1870	0.2333
Village level clusters	Yes	No	No
Village level fixed effects	No	Yes	Yes
Controls	No	No	Yes
Difference: AR vs. RA	0.0165	0.0213	0.0253
Difference: AR vs. RR	0.4838	0.4060	0.3941
Difference: RA vs. RR	0.1912	0.1336	0.1702

* Standard errors in parentheses.

*p ≤ 0.10,

**p ≤ 0.05,

***p ≤ 0.01.

The results are very similar across model specifications. Column (3), our preferred specification, shows that when moving from the fully anonymous condition (AA) to revealing the receiver (AR), mean allocations increase from 1795 Le to 2065 Le (an increase of 15%). Moving from the RA to AR condition statistically increases the mean allocations also (*p* = 0.03). Moving from the AA and RA conditions to the RR condition increases the mean allocation, but the differences are not statistically significant (as standard errors increase).

## Conclusion

Laboratory games may seem contrived and artificial, and it can be difficult to understand how they should apply to the real world. Yet, these games offer the ability to isolate certain human behaviors with a level of precision that is often difficult to do using other methods. In this study, we conduct a set of laboratory exercise to understand how social context impacts behavior in the ultimatum game in small villages in Sierra Leone; i.e., in populations where people know each other well. We find that revealing the identity of receivers increases mean offers, corresponding to findings from laboratory games that measure other social behaviors, like altruism [6] and trust [7], but it has not hitherto been demonstrated for fairness behavior and ultimatum games. These studies suggest that revealing the receiver decreases social distance between sender and receiver, causing senders to increase their offers. In a fairness context, however, even if the social distance between sender and receiver does not change after revealing the receiver, average offers may still increase. This is because the observed allocation in an ultimatum game is presumably a function of what the receiver deems to be fair. Practically, these results imply that a fully anonymous ultimatum game underestimates the expected fair offer. A researcher interested in determining the expected fair offer in social contexts where individuals know each other should, thus, reveal the identity of the receiver. We find no such importance of revealing the identity of the sender on average contributions, although revealing sender and receiver identities in tandem may increase the variance in contributions.

Because the set of factors that matter to senders and receivers are likely to be too complicated for researcher manipulation, full social context can only be introduced into laboratory behavior by revealing identities directly. When the sender and the receiver are not revealed, players must use heuristics like ethnicity and gender, or whatever information is provided by the researcher, to determine behavioral response. The resulting behavior is unlikely to extend to social contexts in which individuals know each other well and behavior does not depend on such heuristics but are a function of nuanced personal factors which result from previous and future real-life interactions among the participants.

In short, the classic ultimatum game abstracts away from important drivers of human behavior such as knowledge of a counterpart’s personal characteristics, previous interactions, and the social networks in which the individuals are embedded. Understanding the influence of this social context is of critical importance if we want to generalize lessons from the lab to real-life settings where individuals know each other well.

## Supporting information

S1 Supporting Information Contains additional information about related studies, the text used in the experiments, and tests for balance across treatment groups. (PDF)

S1 File Code. Contains all necessary code to reproduce results.
(DO) Click here for additional data file.
