# Supplementary material for: Know your neighbor: The impact of social context on fairness behavior
Source: PLoS One. 2018 Apr 11;13(4):e0194037. doi: 10.1371/journal.pone.0194037 (PMC5894984; doi:10.1371/journal.pone.0194037)
Supplement: S1 Supporting Information — (PDF) [file pone.0194037.s001.pdf]

S1 Supporting Information

Know Your Neighbor: The Impact of Social Context on Fairness Behavior

Neelanjan Sircar[1¶], Ty Turley[2¶], Peter van der Windt[3¶*], Maarten Voors[4¶]

[1] Center for Policy Research, New Delhi, India

[2] Marriott School, Brigham Young University, Provo, Utah, United States

[3] Social Science Division, New York University Abu Dhabi, Abu Dhabi, UAE

[4] Development Economics Group, Wageningen University, Wageningen, the Netherlands

* Corresponding author

Email: petervanderwindt@nyu.edu (PW)

¶These authors contributed equally to this work.

Appendix. Related Studies

The table below presents an overview of the fifteen most cited ultimatum game studies in descending order. Based on the search term in Google Scholar "ultimatum game". Accessed: January 14, 2018. [1] and [2] are among the most highly-cited studies but are meta-studies and do not conduct an original ultimatum game experiment. We have therefore left them out of the table.

Table 1: Studies Using the Ultimatum Game

Study	Type	Sender	Receiver	Sample	Participants	Cites
This study	LF	R	R	K	Rural villagers in Sierra Leone	NA
Charness et al. [3]	L	A	A	U	Students at Pompeu Fabra and Berkeley	3,304
Henrich et al. [?]	LF	A	A	K	Members of small-scale societies	2,520
Forsythe et al. [?]	L	A	A	U	Students at University of Iowa	2,090
Falk et al. [4]	L	A	A	U	Unknown	796
Cameron [5]	L	A	A	U	Students in Indonesia	700
Henrich [6]	LF	A	A	K	Small-scale society members and UCLA students	623
Hoffman et al. [7]	L	A	A	U	Subjects at University of Arizona	482
Kagel et al. [8]	L	A	A	U	Undergraduate students	467
Gale et al. [9]	L	A	A	U	Unknown	428
Charness et al. [10]	L	R	A	U	Participants from two universities	412
Bornstein et al. [11]	L	A	A	U	Male students	356
Solnick [12]	L	A	A	U	Students at University of Pennsylvania	354
Solnick et al. [13]	L	R	R	U	Students from two universities	344
Ruffle et al. [14]	L	A	A	U	Subjects recruited via Princeton University	256
Croson [15]	L	A	A	U	Students at Harvard University	219

Column 'Type', "L" indicates laboratory games, whereas "LF" are lab-in-the-field experiments. 'Sender' and 'Receiver', "A" ("R") indicates whether the player is anonymous (revealed) towards the other player. Finally, 'Sample' indicates if the players are known to each other ("K"), or not ("U").

Appendix. Experimental Text

The table below presents the text that was given to the players *verbatim*, after discussing the rules of the ultimatum game.

Table 2: Text by Experimental Condition and Player Type

Condition	Text to Sender	Text to Receiver
AA	"You are a sender. You have 5,000 Le, and you must decide how much to offer your receiver. You can offer any amount from 0 to 5,000 Le. We will NEVER tell you the name of your receiver. We will NEVER tell the receiver your name either. How much do you want to offer the receiver?"	"You are a receiver. You are matched with a sender in your village who could have offered you any amount between 0 and 5,000 Le. You have to decide to accept or reject the offer. We will NEVER tell you the name of your sender. We will NEVER tell the sender your name either. You received an offer of [...] Le. Do you accept or reject the offer?"
AR	"You are a sender. You have 5,000 Le, and you must decide how much to offer your receiver. You can offer any amount from 0 to 5,000 Le. Your receiver is [give the name of the household representative from the household this sender is matched with]. We will NEVER tell the receiver your name. How much do you want to offer the receiver?"	"You are a receiver. You are matched with a sender in your village who could have offered you any amount between 0 and 5,000 Le. You have to decide to accept or reject the offer. We will NEVER tell you the name of your sender. We DID tell the sender your name. You received an offer of [...] Le. Do you accept or reject the offer?"
RA	"You are a sender. You have 5,000 Le, and you must decide how much to offer your receiver. You can offer any amount from 0 to 5,000 Le. We will NEVER tell you the name of your receiver. We WILL tell the receiver your name. How much do you want to offer the receiver?"	"You are a receiver. You are matched with a sender in your village who could have offered you any amount between 0 and 5,000 Le. You have to decide to accept or reject the offer. Your sender is [give the name of the household representative from the household this receiver is matched with]. We will NEVER tell the sender your name. You received an offer of [...] Le. Do you accept or reject the offer?"
RR	"You are a sender. You have 5,000 Le, and you must decide how much to offer your receiver. You can offer any amount from 0 to 5,000 Le. Your receiver is [give the name of the household representative from the household this sender is matched with]. We WILL tell the receiver your name as well. How much do you want to offer the receiver?"	"You are a receiver. You are matched with a sender in your village who could have offered you any amount between 0 and 10,000 Le. You have to decide to accept or reject the offer. Your sender is [give the name of the household representative from the household this receiver is matched with]. We DID tell the sender your name as well. You received an offer of [...] Le. Do you accept or reject the offer?"

Appendix. Balance

The table below lists the average for each variable for all conditions combined and the four different variations, and the difference between them.

Table 3: Descriptive Statistics and Balance Test

Variable	All (sd)	AA (sd)	AR (sd)	RA (sd)	RR (sd)	AA-AR (se)	AA-RA (se)	AA-RR (se)	AR-RA (se)	AR-RR (se)	RA-RA (se)
Sender											
Age	42.59	43.06	44.87	44.46	37.53	-1.80	-1.40	5.53**	0.41	7.33**	6.93**
	(15.05)	(13.44)	(17.53)	(16.15)	(13.85)	(2.94)	(2.15)	(2.45)	(3.71)	(2.98)	(3.01)
Male	0.81	0.86	0.78	0.78	0.77	0.08	0.08	0.09	0.00	0.01	0.00
	(0.39)	(0.35)	(0.42)	(0.42)	(0.42)	(0.08)	(0.06)	(0.07)	(0.07)	(0.06)	(0.06)
# HH members	5.91	5.97	5.93	5.90	5.79	0.03	0.06	0.18	0.03	0.15	0.12
	(2.85)	(2.67)	(2.93)	(2.94)	(3.13)	(0.51)	(0.44)	(0.55)	(0.56)	(0.46)	(0.55)
Potential chief	0.45	0.45	0.46	0.42	0.48	-0.01	0.03	-0.04	0.04	-0.03	-0.07
	(0.50)	(0.50)	(0.50)	(0.50)	(0.50)	(0.09)	(0.09)	(0.08)	(0.10)	(0.08)	(0.08)
Years in village	17.87	17.13	19.40	20.91	15.53	-2.28	-3.79	1.6	-1.51	3.88*	5.39
	(17.02)	(16.68)	(15.30)	(21.42)	(14.10)	(2.69)	(3.29)	(2.61)	(2.80)	(2.04)	(3.25)
Mende	0.88	0.84	0.90	0.90	0.94	-0.06	-0.07	-0.10**	0.00	-0.04	-0.03
	(0.32)	(0.37)	(0.30)	(0.30)	(0.25)	(0.05)	(0.06)	(0.05)	(0.06)	(0.03)	(0.05)
Muslim	0.96	0.94	0.97	0.95	1.00	-0.03	-0.01	-0.06**	0.01	-0.03	-0.05
	(0.20)	(0.24)	(0.18)	(0.21)	(0.00)	(0.03)	(0.04)	(0.03)	(0.04)	(0.02)	(0.03)
Receiver											
Age	44.75	41.31	45.69	44.87	45.54	-4.39**	-3.56	-4.23*	0.82	0.15	-0.67
	(15.14)	(16.02)	(16.02)	(14.19)	(14.47)	(2.05)	(2.64)	(2.32)	(2.16)	(2.32)	(1.91)
Male	0.85	0.79	0.86	0.89	0.83	-0.07	-0.10	-0.03	-0.02	0.04	0.06
	(0.36)	(0.41)	(0.34)	(0.32)	(0.38)	(0.07)	(0.08)	(0.09)	(0.06)	(0.08)	(0.07)
# HH members	6.19	5.49	6.44	6.70	5.78	-0.95	-1.21	-0.29	-0.27	0.66	0.93
	(3.52)	(3.92)	(3.37)	(4.29)	(2.62)	(0.62)	(0.74)	(0.46)	(0.69)	(0.46)	(0.59)
Potential chief	0.46	0.34	0.51	0.53	0.39	-0.17	-0.19**	-0.05	-0.02	0.11	0.14
	(0.50)	(0.48)	(0.50)	(0.50)	(0.49)	(0.12)	(0.09)	(0.16)	(0.09)	(0.08)	(0.13)
Mende	0.85	0.92	0.82	0.93	0.81	0.10	-0.01	0.11	-0.11*	0.01	0.12**
	(0.35)	(0.28)	(0.39)	(0.26)	(0.40)	(0.06)	(0.07)	(0.07)	(0.06)	(0.06)	(0.05)
Muslim	0.96	0.96	0.96	0.98	0.92	0.00	-0.02	0.04	-0.02	0.04	0.06
	(0.20)	(0.20)	(0.19)	(0.14)	(0.27)	(0.03)	(0.03)	(0.05)	(0.03)	(0.04)	(0.04)

Standard errors clustered at the village level. $*p \leq 0.10, **p \leq 0.05, ***p \leq 0.01$.

References

[1] Thaler RH. Anomalies: The Ultimatum Game. Journal of Economic Perspectives. 1988;2(4):195–206.

[2] Oosterbeek H, Sloof R, van de Kuilen G. Cultural Differences in Ultimatum Game Experiments: Evidence from a Meta-Analysis. Experimental Economics. 2004;7(2):171–188.

[3] Charness G, Rabin M. Understanding Social Preferences with Simple Tests. Quarterly Journal of Economics. 2002;117(3):817–869.

[4] Falk A, Fehr E, Fischbacher U. On the Nature of Fair Behavior. Economic Inquiry. 2003;41(1):20–26.

[5] Cameron LA. Raising the Stakes in the Ultimatum Game: Experimental Evidence From Indonesia. Economic Inquiry. 1999;37(1):47–59.

[6] Henrich J. Does Culture Matter in Economic Behavior? Ultimatum Game Bargaining Among the Machiguenga of the Peruvian Amazon. American Economic Review. 2000;90(4):973–979.

[7] Hoffman E, Mccabe KA, Smith VL. On Expectations and the Monetary Stakes in Ultimatum Games. International Journal of Game Theory. 1996;25:289–301.

[8] Kagel JH, Kim C, Moser D. Fairness in Ultimatum Games with Asymmetric Information and Asymmetric Payoffs. Games and Economic Behavior. 1996;13(1):100–110.

[9] Gale J, Binmore KG, Samuelson L. Learning To Be Imperfect: The Ultimatum Game. Games and Economic Behavior. 1995;8:18–20.

[10] Charness G, Gneezy U. What's in a Name? Anonymity and Social Distance in Dictator and Ultimatum Games. Journal of Economic Behavior & Organization. 2008;68(1):29–35.

[11] Bornstein G, Yaniv I. Individual and Group Behavior in the Ultimatum Game: Are Groups More "Rational" Players? Experimental Economics. 1998;1:101–108.

[12] Solnick SJ. Gender Differences in the Ultimatum Game. Economic Inquiry. 2001;39(2):189–200.

[13] Solnick SJ, Schweitzer ME. The Influence of Physical Attractiveness and Gender on Ultimatum Game Decisions. Organizational Behavior and Human Decision Processes. 1999;79(3):199–215.

[14] Ruffle BJ. More Is Better, But Fair Is Fair: Tipping in Dictator and Ultimatum Games. Games and Economic Behavior. 1998;23(2):247–265.

[15] Croson RTA. Information in Ultimatum Games: An Experimental Study. Journal of Economic Behavior & Organization. 1996;30(2):197–212.
